# Fiber–Matrix Interface Engineering in Polymer Composites: Linking Surface Chemistry to Multiscale Mechanical Performance

**DOI:** 10.3390/polym18151842

**Published:** 2026-07-28

**Authors:** Muhammad Tayyab Noman, Nesrine Amor, Musaddaq Azeem, Fiaz Hussain

**Affiliations:** 1 3D Technology Department, Technical University of Liberec, Studentská 1402/2, 461 17 Liberec, Czech Republic; 2 Green Energy and EPC Services, Leicester LE4 9LG, UK; 3 Department of Mechanical Engineering, Gachon University, Seongnam-si 13120, Gyeonggi-do, Republic of Korea

**Keywords:** load transfer mechanisms, interlaminar properties, failure mechanisms, fiber–matrix interface

## Abstract

Fiber–matrix interface engineering plays a decisive role in determining the mechanical performance of fiber-reinforced polymer composites (FRPCs). Despite extensive research on surface modification strategies, translating improvements in interfacial properties into reliable structural performance remains a major challenge. This review critically examines how surface chemistry, interphase architecture, nanomodification, and processing conditions collectively govern load transfer, interfacial shear strength, damage evolution, and overall mechanical behavior. Unlike conventional reviews that primarily summarize modification techniques, this work emphasizes the coupled relationship between interface design and manufacturing, demonstrating that interfacial performance is strongly process-dependent rather than an intrinsic material property. Reported improvements in interfacial metrics are critically evaluated against macroscopic structural performance, revealing persistent limitations arising from dispersion quality, resin rheology, processing defects, and scalability. A multiscale framework is proposed to connect physicochemical modifications with laminate-level failure mechanisms, including delamination, fiber pull-out, and fatigue degradation. The review identifies major research gaps in interphase characterization, standardized evaluation methods, and scalable interface engineering, and outlines future directions based on process-integrated design, predictive modeling, and multifunctional interphases for next-generation composite structures.

## 1. Introduction

**Figure 1:**
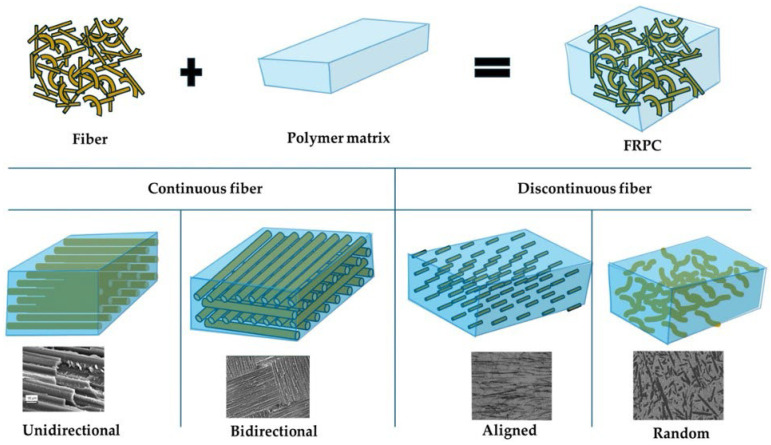
The graphical representation of fiber orientations in FRPCs. Reprinted with permission [20].

FRPCs are widely used in aerospace, automotive, energy, and infrastructure applications due to their high specific strength, stiffness, and design flexibility [1,2,3,4,5,6,7]. However, their structural performance is often limited not by the intrinsic properties of the fiber or matrix, but by the effectiveness of the fiber–matrix interface [8,9,10]. This region governs stress transfer, damage initiation, and failure propagation, making it a critical determinant of composite reliability. The challenge of interface design originates from the fundamental mismatch between reinforcing fibers and polymer matrices in terms of surface energy, chemical reactivity, and mechanical behavior [11,12,13]. In the absence of strong interfacial bonding, load transfer from the matrix to the fibers is inefficient, resulting in premature debonding, fiber pull-out, and interlaminar failure [14,15,16]. As a result, interface engineering has become central to improving composite performance. A wide range of strategies has been developed to enhance interfacial properties, including fiber surface treatments, coupling agents, and nanoscale modifications [17,18,19]. These approaches aim to improve wettability, introduce chemical bonding, and promote mechanical interlocking. FRPCs are generally classified according to fiber length (continuous or discontinuous) and fiber orientation within the polymer matrix as presented in Figure 1.

While many studies report significant increases in interfacial metrics, e.g., interfacial shear strength (IFSS) and interlaminar shear strength (ILSS), their impact on macroscopic properties (tensile strength, fracture toughness, and fatigue resistance) remains inconsistent [21,22,23]. This inconsistency reflects a deeper issue, i.e., interfacial properties are frequently treated as intrinsic material characteristics, whereas in practice, they are strongly dependent on processing conditions. Parameters, e.g., curing kinetics, heat, pressure, and resin infiltration, govern the formation of the interface and can introduce defects including voids and incomplete wetting [24,25,26]. These processing-induced effects often negate the benefits of advanced interface modifications, particularly in nanostructured systems, where dispersion and viscosity constraints limit scalability. In addition, the interphase region, defined as the volume surrounding the fiber with properties distinct from both fiber and matrix, remains insufficiently characterized. Its thickness, composition, and mechanical behavior are difficult to quantify, and are rarely incorporated into predictive models. This limits the ability to establish clear relationships between interfacial modification and structural performance, particularly at the laminate scale where architecture and loading conditions further influence failure mechanisms [27,28]. A comparison of the scope and contributions of representative review articles with the present review is shown in Table 1. As summarized (Table 1), the present review adopts an integrated perspective by critically examining the coupled relationships among interface chemistry, nanomodification, manufacturing processes, and macroscopic failure mechanisms. Rather than providing another descriptive summary of interface modification techniques, this review establishes a multiscale framework that explains why improvements in localized interfacial properties frequently fail to translate into reliable structural performance.

**Figure 2:**
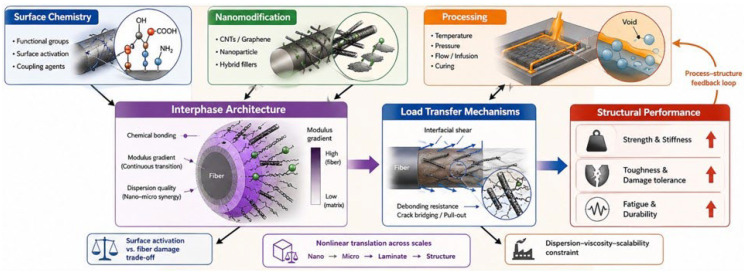
The graphical representation of the multiscale framework.

Accordingly, a critical reassessment of fiber–matrix interface engineering is required. Rather than treating surface chemistry, nanomodification, and processing as independent variables, this review examines their coupled influence on multiscale performance. The central premise is that interfacial improvements are only effective when chemical modification, dispersion, and processing conditions are co-optimized. This review is structured in the following manner. After the introduction and literature selection, Section 3 outlines the fundamental mechanisms governing load transfer and interfacial behavior. Section 4 evaluates surface chemistry approaches, followed by an analysis of nanomodified interfaces in Section 5. Section 6 examines the role of processing in defining interface quality. Section 7 discusses the translation of interfacial properties to macroscopic performance and failure mechanisms followed by Section 8, describing the mechanistic link between interface and structural performance. Finally, Section 9 identifies key research gaps and future directions, and Section 10 provides the concluding remarks. A schematic illustration of our framework is presented in Figure 2.

## 2. Literature Selection

**Figure 3:**
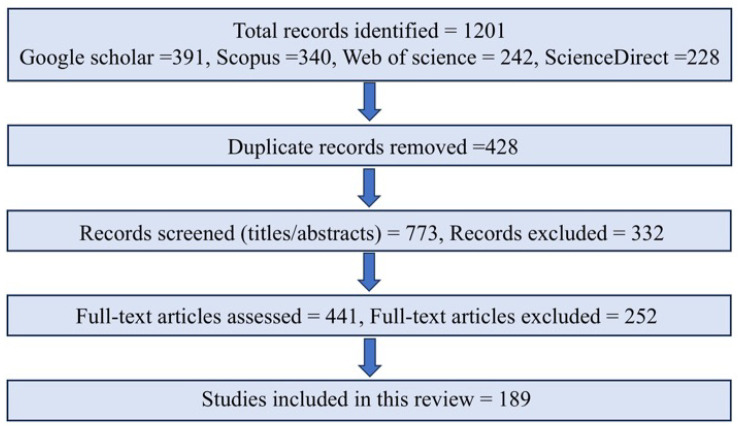
The literature selection criteria from various scientific databases.

This review was developed through a structured and critical evaluation of the contemporary literature on fiber–matrix interface engineering in FRPCs. Relevant publications were primarily identified using the Web of Science, Scopus, and ScienceDirect databases, which collectively provide comprehensive coverage of materials science, polymer engineering, and composite research. The literature search employed combinations of keywords including fiber–matrix interface, surface chemistry, fiber surface treatment, coupling agent, interfacial shear strength, interlaminar shear strength, nanomodification, processing–interface interaction, load transfer, and damage mechanisms. Priority was given to peer-reviewed journal articles published between 2000 and 2026 to capture recent advances in interface engineering. The retrieved literature was critically screened based on its scientific relevance, originality, and contribution to understanding the relationship between interfacial modification and composite performance. Studies focusing solely on bulk matrix or fiber properties without direct relevance to fiber–matrix interactions were excluded. Particular emphasis was placed on investigations reporting the effects of surface treatments, coupling agents, nanostructured interphases, processing conditions, and interphase characterization on interfacial adhesion, stress transfer, damage evolution, and structural mechanical performance. Rather than providing a descriptive summary of published work, the selected literature was comparatively analyzed to identify common trends, conflicting observations, current limitations, and emerging research opportunities. This critical synthesis forms the basis for the multiscale framework proposed in this review, which integrates surface chemistry, processing conditions, and interfacial mechanics to explain their collective influence on the macroscopic performance of FRPC systems. The literature selection methodology is presented in the form of a flow diagram (Figure 3).

## 3. Physics of Load Transfer and Interfacial Mechanics

**Figure 4:**
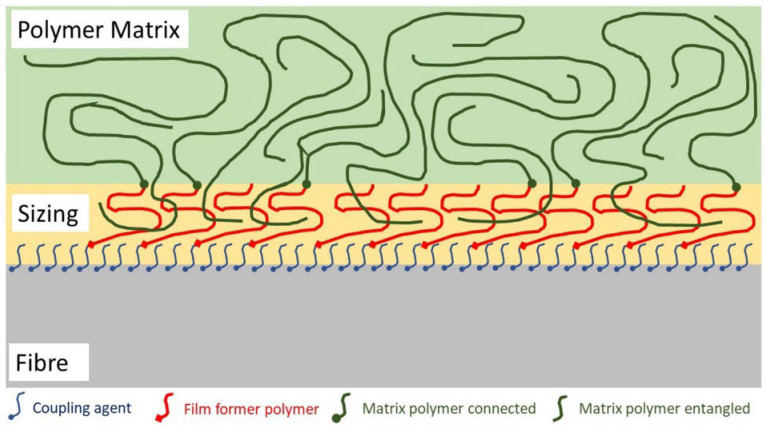
A schematic illustration of the fiber–matrix interface. Reprinted with permission [31].

The mechanical performance of FRPCs is governed by the efficiency of stress transfer between the matrix and the reinforcing fibers. This transfer occurs across the fiber–matrix interface and is primarily controlled by interfacial shear stresses that develop under external loading. The ability of the interface to sustain and transmit these stresses determines whether the composite fails through fiber fracture, matrix cracking, or interfacial debonding. The interfacial adhesion is typically enhanced by the application of a thin coating (sizing). The fiber sizing protects the fibers from abrasion during processing and facilitates handling during compounding and manufacturing. It also enhances the bonding between the fibers and the matrix, thus ensuring a successful stress transfer that inhibits delamination. The role of sizing at the fiber–matrix interface is shown in Figure 4 [31]. It is visible that the coupling agent molecules and film-former polymers are chemically linked to the fiber surface and coupling agent molecules, respectively. The matrix polymers can be either chemically linked (connected) or entangled with film-former polymers.

### 3.1. Shear-Lag Theory and Stress Transfer

Load transfer in discontinuous and continuous fiber composites is commonly described using shear-lag models, which assume that axial stress in the fiber is built up through interfacial shear stresses along the fiber length [32,33,34]. According to this framework, the matrix carries the load initially and transfers it to the fiber through shear at the interface. The efficiency of this process depends on IFSS, fiber aspect ratio, and the elastic properties of both constituents. For short fibers, insufficient interfacial bonding leads to incomplete stress transfer, preventing the fiber from reaching its ultimate tensile strength. The concept of a critical fiber length emerges from this analysis, representing the minimum length required for effective load transfer. Fibers shorter than this length primarily contribute through pull-out mechanisms rather than load bearing, reducing overall composite strength [35,36]. IFSS is widely used as a metric to quantify interface quality. It is typically measured using micromechanical tests, e.g., the single-fiber pull-out test, microbond test, or fragmentation test. While these methods provide useful comparative data, they rely on simplifying assumptions regarding stress distribution and failure modes, which can lead to significant variability in reported values. In addition, IFSS represents a local property that does not fully capture the complexity of stress states in real composite structures [37,38]. In laminated systems, interfacial behavior is influenced by multiaxial loading, residual stresses, and interactions between adjacent plies [39,40]. As a result, increases in IFSS do not necessarily translate into proportional improvements in macroscopic mechanical properties. Although both IFSS and ILSS are widely used to evaluate interface quality in FRPCs, they represent fundamentally different aspects of composite behavior and should not be used interchangeably. IFSS is a micromechanical parameter that quantifies the ability of the fiber–matrix interface to transfer shear stresses at the scale of an individual reinforcing fiber. It is typically determined using single-fiber pull-out, microbond, or fragmentation tests and primarily reflects the local effectiveness of interfacial adhesion and stress transfer. In contrast, ILSS is a laminate-level property measured using short-beam shear tests and characterizes the resistance of adjacent composite plies to interlaminar shear failure. Unlike IFSS, ILSS is influenced not only by fiber–matrix adhesion, but also by laminate architecture, fiber volume fraction, resin properties, void content, residual stresses, and manufacturing defects. Consequently, IFSS provides valuable insight into local interfacial bonding mechanisms, whereas ILSS represents the combined response of the entire composite laminate. An improvement in IFSS therefore does not necessarily produce a proportional increase in ILSS or overall structural performance, highlighting the importance of adopting a multiscale perspective when evaluating interface engineering strategies.

### 3.2. Role of the Interphase Region

The classical assumption of a discrete interface has been replaced by the concept of an interphase, a finite region surrounding the fiber where material properties differ from both the bulk fiber and matrix. This region arises from physicochemical interactions during processing, including diffusion, chemical reactions, and molecular alignment. The mechanical properties of the interphase, including its thickness and modulus gradient, play a critical role in stress transfer and damage evolution [41,42]. The thickness typically ranges from only a few nanometers to several micrometers, depending on the fiber surface treatment, matrix chemistry, and manufacturing process. Unlike the bulk fiber or polymer matrix, the interphase exhibits gradual variations in chemical composition, molecular structure, crosslink density, and mechanical properties rather than a sharp material boundary. Consequently, accurate characterization of the interphase requires complementary analytical techniques capable of resolving both its physicochemical and mechanical gradients across multiple length scales.

A stiff interphase can enhance load transfer but may promote stress concentrations and brittle failure, whereas a more compliant interphase can improve toughness by enabling energy dissipation through plastic deformation. Optimizing interphase properties therefore requires balancing stiffness and ductility. Failure at the fiber–matrix interface typically initiates through debonding when interfacial stresses exceed the local strength [43,44]. This process is influenced by the nature of bonding (mechanical interlocking vs. chemical bonding), surface roughness, and residual stresses introduced during processing. Once debonding occurs, crack propagation may proceed along the interface or deflect into the matrix, depending on the relative toughness of the constituents. Fiber pull-out is a common energy-absorbing mechanism associated with weak or moderately strong interfaces [45,46]. While it contributes to improved toughness, it also indicates inefficient load transfer. Conversely, excessively strong interfaces can suppress pull-out but lead to brittle failure through fiber fracture or matrix cracking. This trade-off highlights that maximizing interfacial strength is not always optimal for overall composite performance.

Interfacial phenomena at the microscale directly influence damage evolution at larger scales [47]. Debonding and matrix cracking at the fiber level can coalesce into interlaminar delamination in laminated composites, ultimately governing structural failure. However, the relationship between microscale interfacial properties and macroscale behavior is non-linear and strongly dependent on composite architecture, loading conditions, and environmental factors [48,49]. Consequently, a multiscale perspective is required to interpret interfacial behavior. Improvements observed in micromechanical tests must be evaluated in the context of laminate-level performance, where competing failure mechanisms and structural constraints determine the ultimate effectiveness of interface engineering strategies.

### 3.3. Characterization Techniques for Interface Engineering

Accurate characterization of the fiber–matrix interface requires a combination of complementary experimental techniques because no single method is capable of fully describing the physicochemical, mechanical, and structural evolution of the interphase. Surface-sensitive analytical techniques, e.g., X-ray photoelectron spectroscopy (XPS), Fourier transform infrared spectroscopy (FTIR), and Raman spectroscopy provide valuable information regarding functional groups, chemical bonding, and surface modification, yet they cannot directly quantify mechanical performance. Conversely, nanoscale methods including atomic force microscopy (AFM) and nanoindentation characterize interphase morphology and local mechanical properties but remain limited by spatial resolution and difficulties associated with isolating extremely thin interphase regions. Micromechanical tests, e.g., single-fiber pull-out, microbond, and fragmentation experiments directly quantify IFSS and provide important insight into local stress-transfer mechanisms [50]. However, these techniques evaluate simplified loading configurations and therefore cannot fully represent the complex stress states encountered in multidirectional composite laminates. At the structural level, ILSS tests provide improved representation of laminate behavior but remain insufficient for describing crack initiation and propagation [51,52]. Consequently, fracture mechanics methods, e.g., double cantilever beam (DCB) and end-notched flexure (ENF) testing have become increasingly important because they directly quantify resistance to delamination under Mode I and Mode II loading conditions, providing stronger correlation with structural durability and service performance [53,54].

Recent developments in in situ characterization techniques, including acoustic emission, digital image correlation (DIC), synchrotron imaging, and X-ray computed tomography (micro-CT), have significantly advanced our understanding of damage initiation and propagation during mechanical loading. Unlike conventional post-mortem microscopy, these methods enable the real-time observation of crack evolution, fiber debonding, and interlaminar failure, thereby bridging the gap between microscale interfacial mechanisms and macroscopic structural behavior [55]. The available evidence therefore indicates that the reliable evaluation of interface engineering requires an integrated characterization strategy combining spectroscopy, microscopy, micromechanical testing, fracture mechanics, and in situ structural monitoring. Table 2 shows the comparison of characterization techniques commonly used in fiber–matrix interface engineering.

## 4. Surface Chemistry for Interface Engineering

Surface chemistry modification remains the most established route for improving fiber–matrix adhesion in polymer composites. These approaches aim to alter the physicochemical characteristics of the fiber surface to enhance wettability, promote chemical bonding, and increase mechanical interlocking with the matrix [83,84]. However, despite decades of development, their effectiveness is highly variable and strongly dependent on processing conditions and material compatibility. This section critically evaluates the principal surface chemistry strategies, with emphasis on their mechanisms, measurable benefits, and practical limitations.

Fiber surface treatments are designed to increase surface energy and introduce functional groups that improve adhesion with polymer matrices. Common methods include oxidative treatments (chemical or electrochemical), plasma treatment, and thermal oxidation [29,85,86]. Oxidative treatments introduce oxygen-containing functional groups (hydroxyl, carbonyl, and carboxyl groups) onto the fiber surface, improving wettability and enabling chemical bonding with reactive matrices (epoxies) [87,88,89]. Plasma treatments achieve similar effects with greater control over surface functionality and minimal environmental impact [90]. These methods can significantly increase IFSS, often by 20–50% under optimized conditions. However, this improvement is not unconditional. Aggressive oxidation can damage the fiber surface, reduce tensile strength, and introduce surface flaws that act as stress concentrators. The trade-off between surface activation and fiber degradation is frequently underreported, leading to the overestimation of treatment effectiveness. In practice, the processing window for optimal treatment is narrow, and small deviations can result in performance loss rather than gain. More importantly, increased surface energy does not guarantee improved composite performance. If resin infiltration is incomplete or curing is suboptimal, the benefits of surface activation are diminished. This reinforces that surface treatment alone cannot ensure effective interface formation.

An alternative to modifying the fiber surface is to tailor the polymer matrix to improve compatibility with the reinforcement. This includes the use of functionalized polymers, reactive diluents, and compatibilizers designed to enhance interfacial bonding [91]. In thermoset systems, matrix functionalization can promote covalent bonding with treated fiber surfaces during curing [92]. In thermoplastic composites, compatibilizers, e.g., maleic anhydride-grafted polymers, are commonly used to improve adhesion with glass or natural fibers [93,94]. These approaches are particularly relevant for recyclable composite systems, where traditional surface treatments may be less effective. While matrix modification can improve interfacial properties, it introduces additional complexity. Changes in polymer chemistry can affect viscosity, curing behavior, thermal stability, and long-term durability [95,96]. As a result, improvements in interfacial adhesion may come at the expense of processability or bulk material performance. In addition, matrix-driven approaches are often less localized than fiber surface treatments, affecting the entire composite system rather than the interface alone. This makes it more difficult to isolate and optimize interfacial effects.

Coupling agents, particularly silanes, are widely used to form covalent or semi-covalent bonds between the fiber surface and polymer matrix [97,98]. These molecules typically contain dual functionality, i.e., one group reacts with the fiber surface, while the other is compatible with or reactive toward the polymer matrix. In glass fiber-reinforced composites, silane coupling agents are highly effective due to the presence of hydroxyl groups on the fiber surface, enabling stable siloxane bonding [99]. This often results in substantial improvements in IFSS and ILSS, along with enhanced resistance to moisture-induced degradation. In carbon fiber systems, however, the effectiveness of coupling agents is less consistent [100]. The chemically inert nature of carbon surfaces limits the formation of strong covalent bonds unless preceded by surface activation [101]. As a result, coupling agents in these systems often rely on secondary interactions, which are weaker and more sensitive to environmental conditions. A critical limitation of coupling agents is their sensitivity to processing and environmental factors. Hydrolysis, incomplete condensation, and non-uniform coatings can lead to weak or heterogeneous interfacial regions [102,103]. Additionally, excessive coupling agent concentration can create a weak boundary layer that reduces load transfer efficiency. These effects are rarely captured in micromechanical testing but become evident at the laminate scale.

Improved wettability is frequently cited as a primary objective of surface modification, as it facilitates resin infiltration and intimate contact between the fiber and matrix. Surface energy measurements and contact angle analysis are commonly used to assess this property [104,105]. However, wettability alone is a poor predictor of interfacial strength. While necessary for effective bonding, it does not account for chemical interactions, interphase formation, or residual stresses. Systems with excellent wettability can still exhibit weak interfaces if chemical bonding is absent or if curing conditions are inadequate. This disconnect highlights a broader issue, i.e., many surface chemistry studies rely on indirect indicators of interface quality rather than direct mechanical performance metrics [106,107]. As a result, improvements in surface energy are often overstated as indicators of composite performance. Despite the wide range of available surface modification techniques, their effectiveness is fundamentally constrained by the following three factors:

- ***Processing dependence:*** Surface modifications only translate into performance gains when compatible with manufacturing conditions, including curing, heat, and resin flow behavior.
- ***Interphase formation:*** The final properties of the interface depend not only on surface chemistry, but also on the structure and properties of the developed interphase region.
- ***Scale transition:*** Improvements in micromechanical properties.

As a result, surface chemistry should be viewed as a necessary but insufficient condition for effective interface engineering. Its role is best understood as one component within a coupled system that includes nanostructuring, processing, and composite architecture. Table 3 presents the improvements in interfacial and mechanical properties achieved by different fiber–matrix interface engineering strategies. The reported values represent typical ranges compiled from representative studies in the literature. The magnitude of improvement depends on fiber type, matrix chemistry, surface treatment conditions, nanomaterial loading, processing route, and testing methodology. Therefore, the reported ranges should be interpreted as indicative rather than absolute values.

## 5. Nanomodified Interfaces

Nanomodification of fiber–matrix interfaces has been widely explored as a strategy to enhance interfacial performance in polymer composites by introducing nanoscale reinforcements, including carbon nanotubes, graphene, nanoclays, and other functional nanomaterials [114,115,116]. These materials offer high aspect ratios and exceptional intrinsic properties, enabling multiple reinforcing mechanisms at the interface. When effectively integrated, nanomaterials can increase surface roughness and promote mechanical interlocking, bridge microcracks and delay their propagation, and provide additional pathways for stress transfer between the matrix and the fiber. As a result, significant improvements in interfacial metrics are reported under controlled conditions. However, these improvements are highly sensitive to the quality of dispersion and the nature of interfacial bonding [30,108]. In the absence of uniform distribution and strong adhesion, nanomaterials can act as defects rather than reinforcements, undermining composite performance. The primary limitation of nanomodification lies in the challenge of achieving stable and uniform dispersion [112]. Due to strong van der Waals interactions, nanomaterials tend to agglomerate during processing, forming clusters that act as stress concentrators and disrupt local stress fields. These agglomerates also interfere with resin flow and reduce effective contact between the fiber and matrix, limiting the formation of a robust interface. Although the surface functionalization of nanomaterials is commonly employed to improve dispersion and compatibility with the polymer matrix, it introduces a trade-off by potentially degrading the intrinsic mechanical or electrical properties of the nanomaterials. Consequently, while low nanomaterial loadings may yield measurable improvements, increasing concentration often leads to diminishing returns or even performance deterioration due to defect accumulation.

Processing constraints further restrict the effectiveness of nanomodified systems. Even at relatively low concentrations, nanomaterials can significantly increase resin viscosity, which adversely affects impregnation during manufacturing processes [111,117]. In an experimental study, resin transfer molding (RTM) was integrated with vacuum infusion to monitor resin flow during the impregnation and infusion processes [118]. Elevated viscosity hinders the complete wetting of fibers, promotes void formation, and can lead to non-uniform distribution of nanomaterials due to filtration effects during flow. These issues are exacerbated in industrial-scale processing, where flow distances are larger and process control is less precise than in laboratory conditions. As a result, nanomodified systems that demonstrate improved interfacial properties in small-scale experiments often fail to maintain these benefits when translated to manufacturing environments. An important distinction in nanomodification strategies lies in whether nanomaterials are introduced locally at the interface or dispersed throughout the bulk matrix. Localized approaches (direct growth or deposition of nanostructures on fiber surfaces) target the interface more efficiently and can enhance load transfer without significantly altering the rheological behavior of the resin [110,113]. In contrast, bulk dispersion modifies the entire matrix, affecting not only interfacial properties, but also processing characteristics, curing behavior, and overall composite performance [109,119]. While bulk approaches are easier to implement, they often dilute the effectiveness of nanomaterials at the interface and introduce unintended trade-offs, whereas localized strategies, although more effective in principle, are more complex and challenging to scale.

A recurring issue in the literature is the inconsistent translation of nanoscale improvements to macroscopic composite performance [120,121]. This discrepancy arises from competing failure mechanisms, non-uniform nanomaterial distribution, and the dominance of processing-induced defects at larger scales. In some cases, excessively strong interfaces resulting from nanomodification can suppress energy-dissipating mechanisms, e.g., fiber pull-out, leading to more brittle failure behavior. These observations highlight that maximizing interfacial strength is not inherently beneficial and must be balanced against the need for toughness and damage tolerance. Despite extensive academic research, the industrial adoption of nanomodified interfaces remains limited. High material costs, challenges in achieving reproducible large-scale processing, health and safety considerations, and the lack of standardized evaluation methods all contribute to this gap. Consequently, many nanomodification strategies remain confined to laboratory-scale demonstrations without clear pathways to commercialization. Nanomodification should be understood as a conditional and context-dependent approach to interface engineering rather than a universal solution. Its effectiveness depends on achieving stable dispersion, maintaining processability, and aligning nanoscale mechanisms with macroscale performance requirements. Without this integration, the addition of nanomaterials introduces complexity without delivering consistent or scalable benefits.

Although nanomodification is widely recognized as an effective approach for enhancing fiber–matrix interfaces, different classes of nanomaterials improve interfacial performance through fundamentally different mechanisms and therefore should not be considered interchangeable. Carbon nanotubes primarily enhance stress transfer through their high aspect ratio, crack-bridging capability, and mechanical interlocking with the surrounding matrix [122,123,124]. However, their effectiveness is frequently limited by agglomeration, increased resin viscosity, and the difficulty of achieving uniform dispersion, particularly in high-fiber-volume laminates. In contrast, graphene and graphene oxide provide exceptionally large specific surface areas that promote interfacial contact and crack deflection while offering additional electrical and thermal functionality [125,126,127]. Nevertheless, graphene-based systems often suffer from nanoplatelet restacking and filtration during liquid composite manufacturing processes, reducing their effectiveness at larger scales. Nanoclays operate through a different reinforcement mechanism by increasing crack tortuosity and improving matrix toughness rather than directly strengthening the fiber–matrix bond [119]. Their relatively low cost and compatibility with conventional polymer processing make them attractive for industrial applications, although complete exfoliation remains difficult to achieve at higher filler loadings. Similarly, silica nanoparticles primarily reinforce the interphase through crack pinning and matrix stiffening while exhibiting better processing compatibility than high-aspect-ratio nanomaterials [128,129]. However, excessive particle loading may increase brittleness and generate local stress concentrations. Hybrid nanomodification strategies attempt to combine the advantages of multiple nanomaterial systems by integrating carbon nanotubes with graphene derivatives or ceramic nanoparticles to create hierarchical stress-transfer pathways. Although these systems frequently report the largest improvements in interfacial properties, they also introduce the greatest challenges regarding processing complexity, reproducibility, quality control, and industrial scalability [130]. Consequently, selecting an appropriate nanomaterial should not be based solely on the maximum improvement in interfacial strength. Instead, the selection should consider the balance between reinforcement mechanism, manufacturability, processing compatibility, durability, cost, and the specific structural requirements of the intended application.

## 6. Processing–Interface Coupling

The properties of the fiber–matrix interface are not solely determined by surface chemistry or material selection, but are fundamentally shaped during composite manufacturing. Processing parameters such as temperature, pressure, curing time, and resin flow behavior govern the formation of the interface and the development of the interphase region [131]. As a result, interfacial properties should not be treated as intrinsic material constants, but as process-dependent outcomes that reflect the combined influence of chemistry, thermodynamics, and manufacturing conditions. One of the most critical aspects of processing–interface coupling is resin infiltration and wetting [132]. Effective load transfer requires intimate contact between the fiber and matrix, which can only be achieved if the liquid resin adequately wets and penetrates the fibers during processing. Parameters, e.g., resin viscosity, fiber energy, and applied pressure, directly influence wetting behavior [133,134,135]. Even when fiber surfaces are chemically treated to enhance wettability, poor control of resin flow can result in incomplete impregnation and the formation of voids or dry spots. These defects act as stress concentrators and significantly reduce interfacial strength, often negating the benefits of prior surface modification. Curing kinetics further complicate interface formation by dictating the evolution of the polymer network and its interaction with the fiber [136,137]. In thermoset systems, the timing and rate of curing reactions determine the extent of chemical bonding at the interface and the development of residual stresses. Rapid curing may trap the system in a non-equilibrium state, leading to incomplete bonding or weak interphase regions, while excessively slow curing can allow for stress relaxation but may compromise productivity and uniformity. The competition between diffusion, reaction, and vitrification processes ultimately controls the structure and properties of the interphase, yet these interactions are rarely optimized in a systematic manner. The formation of a high-quality fiber–matrix interface begins with effective resin infiltration and wetting of the reinforcing fibers. Adequate wetting promotes intimate molecular contact between the fiber surface and polymer matrix, facilitating the development of chemical bonding, mechanical interlocking, and efficient stress transfer across the interface [138,139]. Resin infiltration is governed primarily by surface energy, contact angle, fiber architecture, resin viscosity, and processing pressure. Low-viscosity resin systems generally exhibit improved penetration into fiber bundles, reducing the likelihood of dry spots and incomplete impregnation [140]. Conversely, highly viscous resin systems or densely packed fiber preforms restrict resin flow, producing non-uniform wetting and localized interfacial defects that subsequently become preferred sites for damage initiation. The influence of wetting is therefore not limited to local adhesion but extends to the development of interphase morphology, stress distribution, and structural reliability throughout the composite laminate. Although improved wetting generally enhances interfacial bonding, excessive reduction in resin viscosity is not always desirable because it may increase resin-rich regions, promote filler sedimentation, or adversely affect manufacturing stability. Consequently, resin infiltration represents an optimization problem in which viscosity, processing time, temperature, and fiber architecture must be simultaneously considered to achieve uniform interphase formation without compromising manufacturing efficiency.

Following resin infiltration, the curing process governs the formation and evolution of the interphase. Polymer crosslinking, molecular diffusion, and chemical reactions occurring during curing determine the final thickness, composition, and mechanical properties of the interphase region [141]. Cure temperature, heating rate, dwell time, and degree of cure collectively influence molecular mobility and the establishment of covalent bonds between the fiber surface and surrounding polymer network. Appropriate curing conditions generally improve interfacial adhesion by promoting complete crosslinking and stronger chemical interactions. However, excessively rapid curing or elevated temperatures may reduce molecular diffusion, induce cure shrinkage, and generate residual thermal stresses within the interphase. Similarly, insufficient curing leaves partially reacted polymer chains that reduce interfacial stiffness and long-term durability. Recent investigations further indicate that curing kinetics significantly influence nanomodified systems because nanoparticles modify heat transfer, resin rheology, and local crosslink density [142].

Residual stresses constitute one of the most influential yet frequently underestimated factors governing interface durability. These stresses develop during cooling due to differences in the coefficients of thermal expansion between reinforcing fibers and polymer matrices, combined with polymerization shrinkage occurring during curing [143]. The resulting stress concentrations accumulate within the interphase and may initiate microcracking, interfacial debonding, or matrix damage even before external mechanical loading is applied. The magnitude of residual stresses depends upon the curing temperature, cooling rate, fiber orientation, laminate architecture, and constituent material properties [144]. While higher curing temperatures often improve chemical bonding, they simultaneously increase thermal contraction during cooling, thereby elevating residual stress levels. Similarly, rapid cooling may shorten manufacturing cycles but frequently introduces thermal gradients and localized stress concentrations. Residual stresses also influence crack propagation mechanisms during fatigue loading by accelerating interfacial damage accumulation and reducing resistance to delamination. Their interaction with environmental aging, moisture absorption, and thermal cycling further complicates the prediction of composite durability, demonstrating that interface performance cannot be assessed solely through static mechanical testing. Thermal and mechanical processing conditions also introduce residual stresses that influence interfacial behavior. Differences in the coefficients of thermal expansion between the fiber and matrix generate internal stresses during cooling from processing temperatures. These stresses can either enhance interfacial pressure, improve frictional load transfer, or promote early debonding depending on their magnitude and distribution. Similarly, applied pressure during consolidation affects fiber packing, resin distribution, and void content, all of which contribute to the final quality of the interface. The choice of manufacturing process plays a decisive role in determining how these factors interact. Techniques, e.g., RTM, automatic fiber placement, filament winding, and compression molding impose different constraints on resin flow, temperature gradients, and pressure application [145,146,147]. For example, RTM processes are highly sensitive to resin viscosity and flow dynamics, making them vulnerable to void formation and filtration effects, particularly in nanomodified systems. In contrast, prepreg-based processes offer better control over fiber wetting and resin distribution but introduce challenges related to storage stability and curing uniformity [148]. Consequently, the same interface modification strategy can produce markedly different outcomes depending on the processing route.

An important but often overlooked aspect of processing–interface coupling is the interaction between nanomodification and manufacturability. As discussed in the previous section, the addition of nanomaterials frequently increases resin viscosity and alters flow behavior, which directly impacts impregnation and defect formation. Processing conditions that are adequate for unmodified systems may become unsuitable when nanomaterials are introduced, requiring the re-optimization of temperature, pressure, and flow parameters. Failure to account for this coupling is a major reason why many nanomodified composites fail to achieve consistent performance at larger scales. The influence of processing extends beyond initial interface formation to long-term performance and durability. Variations in curing, residual stress distribution, and defect content affect how the interface responds to cyclic loading, environmental exposure, and aging. Interfaces formed under suboptimal conditions are more susceptible to moisture ingress, thermal degradation, and fatigue-induced debonding, leading to the progressive loss of mechanical integrity over time. These effects are rarely captured in short-term mechanical testing but are critical for structural applications. Therefore, processing should be understood as the controlling variable that integrates material design and interfacial performance. Surface chemistry and nanomodification define the potential for interfacial improvement, but processing determines whether that potential is realized in practice. This perspective shifts interface engineering from a purely materials-focused problem to a manufacturing-driven challenge, where optimization requires the simultaneous consideration of chemistry, processing conditions, and structural requirements.

The influence of manufacturing parameters on fiber–matrix interface quality should not be interpreted as independent or universally beneficial. Instead, interface development results from the coupled interaction of curing conditions, consolidation pressure, resin rheology, fiber architecture, and defect formation, all of which simultaneously influence interphase evolution and stress transfer. Increasing the curing temperature generally promotes polymer crosslinking and chemical bonding at the interface; however, excessive curing temperatures may generate residual thermal stresses, matrix shrinkage, and brittle interphase behavior that reduce fracture resistance [149,150]. Similarly, higher consolidation pressures improve fiber wetting and reduce void content but may also induce fiber distortion or excessive resin squeeze-out when applied beyond the optimum processing window [151,152]. Resin rheology represents another critical factor because sufficiently low viscosity facilitates fiber impregnation and interphase formation, whereas excessive viscosity restricts resin infiltration and promotes dry spots and interfacial defects [153,154]. In addition, although increasing the fiber volume fraction generally improves composite stiffness and load-bearing capacity, excessive fiber packing may reduce resin accessibility and hinder complete wetting, thereby increasing stress concentrations and reducing interfacial reliability [155,156]. These examples demonstrate that processing–interface coupling should be viewed as a multidimensional optimization problem in which competing mechanisms determine the final mechanical performance of the composite.

## 7. From Interfacial Metrics to Structural Performance

The ultimate objective of fiber–matrix interface engineering is not the improvement of micromechanical parameters, but the enhancement of structural performance at the composite level. However, establishing a direct and consistent relationship between interfacial properties (IFSS or ILSS) and macroscopic mechanical behavior remains a persistent challenge. This disconnect reflects the complex and nonlinear nature of damage evolution in composite materials. One of the primary reasons for this inconsistency is the presence of competing failure mechanisms [157,158]. In an ideal system with optimized interfacial properties, load is efficiently transferred to the fibers, leading to fiber-dominated failure and maximized strength. However, when the interface is weak, failure occurs through debonding and fiber pull-out, resulting in reduced strength but potentially increased energy absorption. Conversely, excessively strong interfaces can suppress these energy-dissipating mechanisms, promoting brittle failure through matrix cracking or fiber fracture [159,160]. As a result, there exists an optimal range of interfacial strength that balances load transfer efficiency with damage tolerance, rather than a simple objective of maximizing adhesion. The influence of interfacial properties becomes even more complex at the laminate scale, where interactions between plies govern structural behavior [161,162]. Interlaminar stresses generated under bending, impact, or torsional loading can lead to delamination, a dominant failure mode in many composite structures [163,164,165]. While improvements in ILSS are often cited as evidence of enhanced interfacial performance, these measurements do not fully capture resistance to delamination propagation, which depends on fracture toughness and energy release rates. Consequently, composites with higher ILSS may still exhibit poor resistance to crack growth if the interphase lacks sufficient toughness. Table 4 summarizes representative studies reporting simultaneous measurements of interfacial properties and structural performance. Although improvements of 20–80% in IFSS are frequently reported, corresponding increases in tensile strength rarely exceed 10–30%, and fatigue performance remains strongly dependent on processing quality, laminate architecture, and defect population. Large improvements in IFSS frequently produce only moderate improvements in tensile strength because structural performance is controlled simultaneously by interphase toughness, processing quality, void content, laminate architecture, and damage evolution.

Fatigue behavior further illustrates the limitations of relying on static interfacial metrics. Under cyclic loading, damage accumulates gradually through matrix cracking, interfacial debonding, and delamination growth [166,167,168,169]. The rate and path of damage evolution depend not only on interfacial strength, but also on interfacial properties, residual stresses, and the presence of processing-induced defects. Interfaces optimized for static strength may degrade rapidly under fatigue conditions if they are unable to accommodate repeated stress redistribution. This highlights the need to evaluate interfacial performance under realistic loading scenarios rather than relying solely on quasi-static tests. Environmental factors introduce additional complexity in the translation of interfacial properties. Exposure to moisture, temperature fluctuations, and chemical environments can degrade interfacial bonding through mechanisms (hydrolysis, plasticization of the matrix, and thermal mismatch stresses) [170,171]. Interfaces that rely primarily on physical interactions or weak chemical bonds are particularly susceptible to such degradation. Even systems with initially high interfacial strength may experience significant reductions in performance over time, undermining long-term reliability.

**Figure 5:**
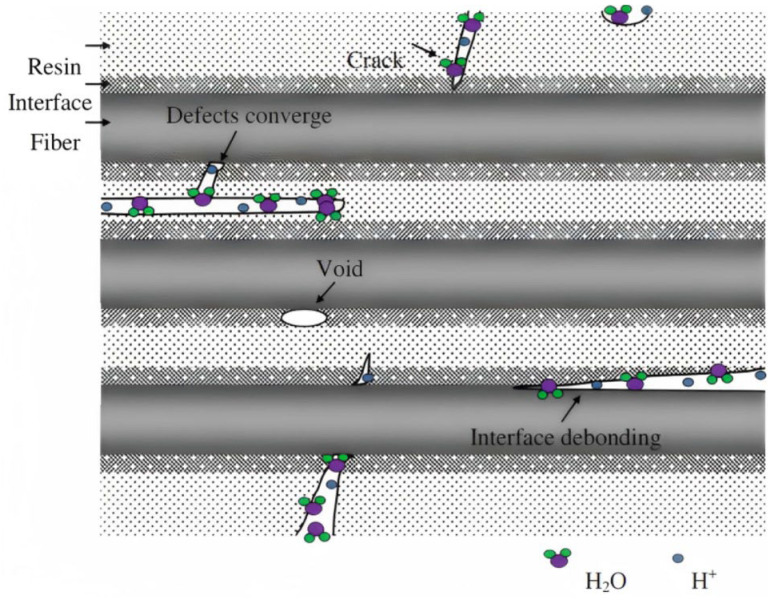
A representation of cracks, interface debonding, and voids in FRPCs. Reprinted with permission [170].

Another critical factor is the role of defects introduced during processing. Voids, incomplete wetting, fiber misalignment, and non-uniform interphase formation can dominate failure behavior at the structural level. In many cases, the impact of these defects outweighs the benefits of improved interfacial chemistry or nanomodification [172]. This explains why composites with similar interfacial treatments can exhibit markedly different performance depending on manufacturing quality. It also reinforces the argument that interfacial engineering cannot be decoupled from processing considerations. The lack of direct correlation between interfacial metrics and structural performance also reflects limitations in current characterization methods [173,174]. Techniques used to measure IFSS or ILSS often involve simplified stress states and idealized conditions that do not represent real conditions. As a result, these metrics provide only partial insight into interfacial behavior and may lead to misleading conclusions when used as sole indicators of performance. Therefore, a more comprehensive evaluation integrating micromechanical testing with fracture mechanics approaches and laminate-level characterization is required for a better understanding. A schematic of defects in FRPCs is shown in Figure 5.

Ultimately, the translation of interfacial properties to macroscopic performance is governed by a combination of factors, including interface strength, interphase toughness, composite architecture, processing quality, and loading conditions. Improvements at the microscale must be interpreted within this broader context to assess their true impact. This perspective challenges the common assumption that enhancing interfacial properties will automatically lead to better composite performance, emphasizing instead the need for a balanced and system-level approach. In this context, effective interface engineering should aim not only to maximize adhesion, but to tailor interfacial behavior to the requirements of specific applications. For load-bearing structures, this may involve optimizing the balance between strength and toughness to delay failure and improve damage tolerance. For fatigue-critical applications, the focus may shift toward resistance to crack initiation and propagation under cyclic loading. Recognizing these distinctions is essential for translating interfacial design into meaningful performance gains in real-world composite systems. Table 5 represents the relationship between commonly used interfacial characterization metrics and structural performance in FRPCs.

## 8. Mechanistic Pathway Linking Interface Engineering to Structural Performance

The multiscale framework proposed in this review is not intended as a new predictive model but rather as an evidence-based synthesis of experimentally and numerically validated relationships reported throughout the literature. A consistent observation across studies is that modifications introduced at the molecular scale propagate through multiple hierarchical length scales before influencing the macroscopic behavior of FRPCs. Consequently, understanding structural performance requires consideration of the complete sequence of physicochemical, micromechanical, and structural processes rather than isolated improvements in individual interfacial parameters [177]. The propagation pathway begins with surface chemistry modification, where oxidation, plasma treatment, coupling agents, or nanomaterial functionalization alter the chemical composition, surface energy, and wettability of reinforcing fibers. These modifications are commonly verified using XPS, FTIR, Raman spectroscopy, and contact-angle measurements, which demonstrate the formation of oxygen-containing functional groups, improved chemical compatibility, and enhanced surface reactivity. Such physicochemical changes promote stronger chemical bonding and mechanical interlocking between the fiber and polymer matrix while simultaneously influencing resin wetting and interphase formation [178,179,180]. The modified surface chemistry subsequently governs the development of the interphase, a finite transition region possessing mechanical and chemical characteristics distinct from both the fiber and the bulk polymer matrix. Experimental investigations employing AFM, nanoindentation, TEM, and SEM have demonstrated that interphase thickness, stiffness, and morphology strongly affect local stress distribution and crack initiation. Rather than functioning as an abrupt interface, the interphase acts as a stress-transfer zone that redistributes mechanical loads and controls the initiation of interfacial damage under external loading. Changes within the interphase are reflected in micromechanical properties, particularly IFSS, which is commonly measured using single-fiber pull-out, microbond, or fragmentation tests. Numerous studies report substantial improvements in IFSS following surface functionalization or nanomodification, confirming enhanced local load-transfer efficiency [181,182,183]. However, as demonstrated throughout this review, IFSS represents only the local response of an individual fiber–matrix interface. Its improvement alone cannot guarantee proportional enhancement of structural performance because damage evolution within composite laminates is governed by additional mechanisms including matrix cracking, fiber bridging, crack deflection, residual stresses, void formation, and ply interactions.

As damage progresses beyond the microscale, the dominant mechanisms transition toward interlaminar fracture and delamination, where fracture toughness rather than local interfacial adhesion becomes the governing performance indicator. DCB and ENF tests have consistently shown that Mode I and Mode II interlaminar fracture toughness provide more representative assessments of resistance to crack initiation and propagation under realistic structural loading conditions. These fracture parameters integrate the combined effects of interface quality, interphase architecture, matrix deformation, fiber bridging, and crack-path evolution, thereby providing a stronger correlation with structural reliability than IFSS or ILSS alone. The final stage of the propagation pathway occurs at the laminate and structural scale, where composite performance is influenced simultaneously by interface quality, laminate architecture, manufacturing defects, environmental degradation, and loading history. Tensile strength, compressive strength, impact resistance, fatigue life, and long-term durability therefore emerge from the interaction of multiple hierarchical mechanisms rather than from interfacial adhesion alone. Experimental studies have repeatedly demonstrated that significant improvements in local interfacial properties frequently translate into only moderate structural gains when factors such as resin viscosity, fiber impregnation, curing conditions, residual stresses, or void content limit efficient stress transfer throughout the laminate [184,185,186]. This observation explains why apparently successful interface modifications at the laboratory scale often exhibit reduced effectiveness during industrial manufacturing. The collective evidence reviewed in this work therefore supports a hierarchical cause-and-effect pathway in which molecular-scale surface chemistry governs interphase formation, the interphase determines local stress-transfer behavior, local stress transfer influences damage initiation, damage evolution controls fracture and delamination resistance, and these mechanisms ultimately dictate laminate-scale structural performance. Importantly, each stage is experimentally supported through complementary characterization techniques, including spectroscopy, microscopy, micromechanical testing, fracture mechanics, and full-scale mechanical evaluation. Table 6 shows the results supporting multiscale interface–property relationships.

## 9. Critical Gaps and Future Directions

Despite extensive progress in fiber–matrix interface engineering, several fundamental limitations continue to restrict the ability to achieve consistent and scalable improvements in composite performance. These limitations are not isolated issues, but systemic gaps that arise from how interface design is currently approached, often in fragmented, chemistry-driven ways that neglect processing and multiscale behavior. Addressing these gaps requires a shift from incremental modification strategies toward integrated and application-driven frameworks. A primary challenge lies in the lack of standardized and reliable methods for characterizing the interphase region. While IFSS and ILSS are widely used as indicators of interface quality, they provide only indirect and often incomplete representations of interfacial behavior. The interphase, which governs stress transfer and damage evolution, remains difficult to isolate and quantify experimentally due to its slender thickness and spatial variability. As a result, its mechanical properties are often inferred rather than directly measured, limiting the accuracy of predictive models and hindering the development of design guidelines.

Another critical gap is the weak and inconsistent correlation between microscale interfacial improvements and macroscopic composite performance. This disconnect reflects the absence of validated multiscale frameworks that can link interfacial phenomena to laminate-level behavior under realistic loading conditions. Without such frameworks, interface engineering remains largely empirical, relying on trial-and-error approaches rather than predictive design. Scalability represents a persistent barrier, particularly for advanced interface modification strategies involving nanomaterials. While nanomodified interfaces can demonstrate significant improvements in controlled laboratory environments, their implementation in industrial processes is constrained by issues, e.g., dispersion, viscosity increase, process compatibility, and cost [187]. The lack of reproducibility at larger scales further limits their practical adoption. Consequently, many proposed solutions remain technologically promising but industrially unviable.

Processing–interface coupling remains insufficiently addressed in current research. Interface properties are often optimized independently of manufacturing conditions, despite clear evidence that processing parameters govern interphase formation, defect generation, and residual stress development. This disconnect leads to situations where interface modifications that are effective under idealized conditions fail during actual manufacturing. Future work must therefore integrate processing considerations into the design of interface engineering strategies, rather than treating them as secondary factors. Durability and environmental stability also require greater attention. Many interface modification techniques are evaluated based on short-term mechanical performance, with limited consideration of long-term behavior under environmental exposure. Moisture absorption, thermal cycling, and chemical degradation can significantly alter interfacial properties over time, particularly in systems relying on weak chemical or physical interactions [188,189]. The absence of standardized long-term testing protocols further complicates the assessment of durability, making it difficult to compare results across studies.

Looking forward, advancing the field of interface engineering will require a transition toward integrated, multiscale, and data-driven approaches. One key direction is the development of predictive models that couple surface chemistry, processing conditions, and mechanical behavior across length scales. Such models should incorporate realistic representations of the interphase and account for variability introduced during manufacturing. The integration of experimental data with computational techniques, including machine learning, offers potential for accelerating this process, although current applications remain limited in scope. Another important direction is the design of multifunctional interphases that extend beyond mechanical performance. By incorporating electrical, thermal, or self-sensing capabilities at the interface, it is possible to enable new functionalities, e.g., structural health monitoring and energy storage. However, achieving this without compromising mechanical integrity or processability remains a significant challenge, requiring careful balancing of competing requirements.

From a manufacturing perspective, emphasis should be placed on developing scalable and process-compatible interface modification techniques. This includes approaches that minimize changes to resin rheology, enable uniform application at industrial scales, and maintain cost-effectiveness. Localized modification strategies (controlled fiber surface functionalization) may offer advantages over bulk approaches if they can be reliably implemented in continuous processing environments. Ultimately, the future of fiber–matrix interface engineering depends on redefining the problem from one of material enhancement to one of system optimization. Rather than seeking maximum interfacial strength or isolated property improvements, the focus must shift toward achieving robust, reproducible, and application-specific performance. This requires aligning material design, processing conditions, and structural requirements within a unified framework. Without this shift, further advances in surface chemistry or nanomodification are unlikely to produce meaningful gains in real-world composite systems. Table 7 presents the principles for next generation FRPCs.

## 10. Integrative Framework and Concluding Remarks

Fiber–matrix interface engineering has evolved from conventional surface treatment strategies toward integrated interphase design involving surface chemistry, nanomodification, processing optimization, and multiscale characterization. However, the literature reviewed in this work demonstrates that improvements in local interfacial properties do not automatically translate into superior structural performance. While numerous studies report significant increases in IFSS and ILSS, the resulting improvements in tensile strength, fracture toughness, fatigue life, and long-term durability remain highly dependent on processing quality, interphase architecture, defect formation, and loading conditions. Consequently, interface engineering should no longer be viewed as an isolated materials problem, but as a systems-engineering challenge that requires the simultaneous optimization of material chemistry, manufacturing processes, and structural mechanics.

A central conclusion emerging from this review is that the objective of interface engineering is not to maximize interfacial adhesion, but to optimize competing performance requirements. An excessively weak interface results in inefficient load transfer, premature debonding, and reduced structural strength, whereas an excessively strong interface suppresses beneficial energy-dissipation mechanisms (controlled fiber pull-out and crack deflection), often leading to brittle failure. The optimum interface therefore represents a carefully balanced interphase capable of providing efficient stress transfer while simultaneously maintaining fracture toughness, fatigue resistance, and damage tolerance. This balance becomes increasingly important in advanced composite structures subjected to cyclic loading, impact, thermal cycling, and aggressive environmental conditions, where long-term reliability is governed by the interaction between interfacial properties and processing-induced defects rather than by interfacial strength alone.

Another important finding is that processing conditions are equally important as surface modification strategies in determining final interface quality. Parameters, e.g., resin viscosity, curing kinetics, consolidation pressure, temperature history, fiber wetting, and void formation, directly influence the development of the interphase and ultimately determine whether laboratory-scale improvements can be translated into industrial manufacturing. This explains why many nanomodification strategies demonstrating excellent micromechanical performance fail to deliver comparable structural benefits at larger scales. Future interface design should therefore integrate chemistry, processing, and structural design within unified optimization frameworks instead of treating them as independent variables.

Based on the critical assessment presented throughout this review, several technical benchmarks should guide future interface engineering. First, interface optimization should simultaneously improve interfacial strength and fracture toughness rather than maximizing either property individually. Second, interface modifications should demonstrate reproducible performance after long-term fatigue loading, moisture exposure, thermal cycling, and environmental aging. Third, advanced interface technologies should remain compatible with scalable manufacturing processes while minimizing resin viscosity increase, nanomaterial agglomeration, and processing defects. Finally, future evaluation should extend beyond IFSS and ILSS measurements by incorporating fracture mechanics, durability assessment, and laminate-scale mechanical characterization to establish reliable relationships between microscale interfacial phenomena and structural performance.

The following research priorities are proposed to accelerate the development of next-generation FRPCs:

- Develop standardized multiscale characterization protocols that combine IFSS, ILSS, fracture toughness, nanoindentation, spectroscopy, and microscopy to provide a comprehensive description of the interphase.
- Establish predictive multiscale computational models capable of linking surface chemistry, interphase evolution, processing conditions, and structural failure under realistic service environments.
- Design multifunctional interphases that simultaneously provide mechanical reinforcement, thermal management, electrical conductivity, structural health monitoring, and self-sensing capabilities without compromising manufacturability.
- Integrate artificial intelligence, machine learning, and digital twins into interface design and manufacturing optimization to accelerate materials discovery, process optimization, and performance prediction.
- Develop scalable and industrially compatible interface engineering technologies suitable for automated manufacturing processes, including RTM, automated fiber placement, filament winding, additive manufacturing, and compression molding.
- Establish application-oriented design criteria that explicitly balance strength, fracture toughness, fatigue resistance, environmental durability, manufacturability, sustainability, and cost according to the specific requirements of aerospace, automotive, wind energy, marine, and hydrogen-storage applications.

The future of fiber–matrix interface engineering will not be defined by developing stronger interfaces alone, but by designing interfaces that achieve the optimum balance between load-transfer efficiency, damage tolerance, fatigue resistance, long-term durability, manufacturability, and economic viability.

## Figures and Tables

**Table 1:** Comparison of the present review with representative recent reviews on fiber–matrix interface engineering.

Primary Focus	Surface Chemistry	Nanomodification	Processing–Interface Coupling	Multiscale Performance	Comparison of Interface Strategies	References
Interface and interphase concepts	✔	✗	✗	Partial	✗	[9]
Carbon fiber interface characterization	✔	Partial	✗	Partial	✗	[10]
Natural fiber surface modification	✔	✗	✗	Limited	✗	[13]
Surface modification of aramid fibers	✔	Partial	✗	Limited	✗	[17]
Surface activation of polymer fibers	✔	✗	Limited	Limited	✗	[29]
Dispersion and interfacial adhesion of nanofillers	Partial	✔	Partial	Limited	Partial	[30]
Interface characterization methods	Partial	✗	✗	Partial	✗	[12]
**Integrated interface engineering framework**	**✔**	**✔**	**✔**	**✔**	**✔**	**Present review**

**Table 2:** Critical comparison of characterization techniques used in fiber–matrix interface engineering.

Technique	Primary Measurement	Length Scale	Major Advantages	Major Limitations	Typical Applications	References
XPS	Surface elemental composition and functional groups	Molecular	High chemical sensitivity	Very shallow penetration; no mechanical information	Surface functionalization, oxidation, coupling-agent analysis	[56,57]
FTIR	Chemical bonding and functional groups	Molecular	Rapid, non-destructive	Limited spatial resolution	Surface chemistry verification	[58,59]
Raman spectroscopy	Chemical structure, defects, residual stress	Molecular–Microscale	Stress mapping, carbon characterization	Fluorescence interference; local measurement	CNTs, graphene, carbon fibers	[60,61]
AFM	Surface topography and nanoscale modulus	Nanoscale	High spatial resolution	Small scan area; time-consuming	Interphase thickness, nanoscale mechanics	[62,63]
Nanoindentation	Local hardness and elastic modulus	Nanoscale	Quantitative mechanical properties	Difficult to isolate thin interphase	Interphase mechanical characterization	[64,65]
Scanning electron microscopy (SEM)	Fracture morphology and failure mechanisms	Microscale	Excellent visualization of damage	Mainly qualitative	Fiber pull-out, crack propagation, void analysis	[66,67,68]
Transmission electron microscopy (TEM)	Interphase nanostructure	Nanoscale	Atomic-scale structural information	Complex sample preparation	Interphase morphology	[69,70]
Single-fiber pull-out/Microbond	IFSS	Microscale	Direct assessment of local stress transfer	Simplified loading condition	Interface optimization	[71,72]
Short-beam shear	ILSS	Laminate	Simple and standardized	Does not directly measure fracture behavior	Laminate quality assessment	[73,74]
DCB (Mode I)	GIc	Laminate	Direct fracture toughness measurement	Specimen preparation sensitive	Delamination resistance	[75,76]
ENF (Mode II)	GIIc	Laminate	Shear fracture characterization	More complex analysis	Fatigue and shear delamination	[77,78]
Acoustic emission/DIC	In situ damage evolution	Structural	Real-time damage monitoring	Data interpretation complexity	Damage initiation and progression	[79,80]
Micro-CT	Internal defects and voids	Microscale–Laminate	Non-destructive 3D imaging	High equipment cost	Void analysis, damage evolution	[81,82]

**Table 3:** Representative improvements in interfacial and mechanical properties.

Interface Engineering Strategy	Typical Improvement in IFSS (%)	Typical Improvement in ILSS (%)	Typical Improvement in Tensile/Flexural Properties (%)	Mechanism	Limitations	References
Chemical oxidation (acid/electrochemical)	15–45	10–30	5–20	Introduction of oxygen-containing functional groups and increased surface energy	Fiber surface damage under severe oxidation	[85,86]
Plasma treatment	20–60	15–40	10–25	Surface activation without affecting bulk fiber properties	Treatment effectiveness depends strongly on processing parameters	[83,90]
Thermal oxidation	10–35	10–25	5–15	Increased surface roughness and chemical activation	Narrow processing window; excessive oxidation degrades fibers	[29]
Silane coupling agents	25–70	20–50	10–30	Covalent bonding between fiber surface and polymer matrix	Sensitive to hydrolysis and processing conditions	[97,99,102]
Maleic anhydride compatibilizers (thermoplastics)	20–50	15–35	10–25	Improved chemical compatibility between fibers and matrix	Changes matrix rheology and processing behavior	[91,93]
Carbon nanotubes	30–80	20–60	10–35	Crack bridging, mechanical interlocking, additional load-transfer pathways	Agglomeration, viscosity increase, high cost	[108,109]
Graphene/graphene oxide	25–70	20–50	10–30	Increased interfacial area and crack deflection	Difficult dispersion and filtration during processing	[110,111]
Hybrid nanomodification	35–90	30–70	15–40	Synergistic reinforcement and multiscale stress transfer	Complex processing and limited industrial scalability	[112,113]

**Table 4:** The selected studies illustrating the relationship between interfacial properties and structural performance in FRPCs.

IFSS Improvement (%)	ILSS Improvement (%)	Fracture Toughness	Tensile/Flexural Improvement	Fatigue Performance	Main Conclusion	References
↑	↑	↑	Moderate	Improved	Structural gains depend on laminate architecture	[161]
↑↑	↑	↑↑	Moderate	Improved	Optimized interphase more effective than maximum adhesion	[41]
↑↑	Moderate	Moderate	Small	Limited	Dispersion controls structural benefits	[109]
↑	—	↑	Moderate	Improved	Toughness improved more than strength	[108]
↑	↑	Moderate	Small	—	High viscosity limits structural performance	[111]

**Table 5:** Relationship between commonly used interfacial characterization metrics and structural performance in FRPCs.

Interfacial Metric	Common Test Method	Structural Property Primarily Affected	Correlation with Structural Performance	Limitations	References
**IFSS**	Single-fiber pull-out, microbond, fragmentation test	Tensile strength, load-transfer efficiency	Moderate	Localized measurement; simplified stress state; does not represent laminate-scale behavior	[8,37,173]
**ILSS**	Short-beam shear test (ASTM D2344)	Delamination resistance, interlaminar load transfer	Moderate–High	Does not directly predict crack initiation or propagation; sensitive to specimen geometry	[21,22,23]
**Mode I interlaminar fracture toughness**	DCB	Resistance to delamination initiation and propagation	High	Strong dependence on specimen preparation, laminate architecture, and crack length measurement	[161,163]
**Mode II interlaminar fracture toughness**	ENF	Fatigue resistance and shear-driven delamination	High	Sensitive to loading configuration, laminate stacking sequence, and fiber orientation	[162,165]
**Interphase modulus/hardness**	Nanoindentation, AFM	Local stress transfer, crack initiation	Moderate	Difficult to isolate the thin interphase; high spatial variability	[42,110]
**Surface chemistry**	XPS, FTIR, Raman spectroscopy	Chemical bonding, wettability, interface quality	Indirect	Indicates chemical modification but cannot directly predict mechanical performance	[175,176]
**Interfacial morphology**	SEM, TEM	Failure mechanism, fiber pull-out, crack propagation	Qualitative	Mainly post-failure characterization; limited quantitative correlation	[43,44]

**Table 6:** Experimental and numerical evidence supporting multiscale interface–property relationships.

Scale	Property	Measurement	Structural Implication	References
Molecular	Functional groups	XPS, FTIR	Chemical bonding	[29,97]
Interphase	Modulus, thickness	AFM, Nanoindentation	Stress distribution	[41,42]
Microscale	IFSS	Pull-out	Load transfer	[8,173]
Mesoscale	GIc/GIIc	DCB, ENF	Delamination	[161,162,163]
Laminate	Tensile/Fatigue	ASTM tests	Structural integrity	[21,22,23]

**Table 7:** Design principles for next-generation fiber–matrix interface engineering in FRPCs.

Design Objective	Desired Outcome	Engineering Challenge	Recommended Strategy
Strength	Efficient load transfer	Fiber debonding	Controlled chemical bonding
Toughness	Crack deflection	Brittle fracture	Optimized interphase modulus
Fatigue resistance	Slow damage accumulation	Cyclic debonding	Toughened interphase
Processability	Uniform impregnation	High resin viscosity	Localized interface modification
Durability	Moisture and thermal stability	Interphase degradation	Stable coupling chemistry
Scalability	Industrial manufacturing	Nanomaterial dispersion	Process-integrated interface design

## Data Availability

The original contributions presented in this study are included in the article. Further inquiries can be directed to the corresponding authors.
